# Improving stress management, anxiety, and mental well-being in medical students through an online Mindfulness-Based Intervention: a randomized study

**DOI:** 10.1038/s41598-023-35483-z

**Published:** 2023-05-22

**Authors:** Teresa Fazia, Francesco Bubbico, Andrea Nova, Chiara Buizza, Herald Cela, Davide Iozzi, Beril Calgan, Federica Maggi, Valentina Floris, Irene Sutti, Salvatore Bruno, Alberto Ghilardi, Luisa Bernardinelli

**Affiliations:** 1 Department of Brain and Behavioral Sciences, University of Pavia, Pavia, Italy; 2 Department of Clinical and Experimental Sciences, University of Brescia, Brescia, Italy; 3 University of Graz, Graz, Austria; 4 Istituto di Psicosintesi, Milan, Italy

**Keywords:** Psychology, Human behaviour

## Abstract

Pressures and responsibilities of medical school put a strain on medical student's personal wellbeing, leading among all to high rates of anxiety, emotional discomfort and stress. In this work we evaluated the effectiveness of a comprehensive Mindfulness-Based Intervention (MBI) in reducing this load. The intervention comprised 10 twice-a-week Integral Meditation classes, dietary advice, and brief yoga sessions. We performed a randomized trial on two cohort of medical students from Italian universities: 239 in cohort 1 (106 treated and 133 controls), and 123 in cohort 2 (68 treated and 55 control) for a total sample of 362 students. Nine questionnaires for evaluating the effectiveness of our intervention on stress (PSS), state anxiety (STAIX-1), well-being (WEMWBS), mind-wandering (MW-S), overall distress (PANAS), emotion regulation (DERS), resilience (RS-14), and attentional control (ACS-C and ACS-D) were collected both pre and post intervention. Linear mixed effect models were run on the whole sample showing that, after multiple testing correction, our intervention was effective in reducing perceived stress (β = − 2.57 [− 4.02; − 1.12], p = 0.004), improving mental well-being (β = 2.82 [1.02; 4.63], p = 0.008) and emotional regulation (β = − 8.24 [− 12.98; − 3.51], p = 0.004), resilience (β = 3.79 [1.32; 6.26], p = 0.008), reducing the tendency to wander with the mind (β = − 0.70 [− 0.99; − 0.39], p = 0.0001), ameliorating the ability to maintain attention (AC-S (β = − 0.23 [− 0.44; − 0.02], p = 0.04) and AC-D (β = − 0.19 [− 0.36; − 0.01], p = 0.04)), and the overall distress (β = 1.84 [0.45; 3.23], p = 0.02).

## Introduction

In the scientific literature, the responsibilities and pressures of medical school and residency are widely known for putting a strain on medical student's personal wellbeing, leading to high rates of anxiety, depression, burnout, and emotional discomfort^1^. Academic pressure^2^, financial concerns, workload, sleep deprivation^3^, drugs abuse^4^, exposure to patients' suffering and fatalities^5,6^ are among the numerous causes linked to such a decrease in mental well-being in medical studies setting. The so-called *hidden curriculum* is also a factor considered in medical education. It is based on implicit teaching that includes moral and ethical values among medical education. When this teaching goes in a negative direction such as cynicism towards patients, future career, or competition with colleagues, can become one of the causes influencing psychological distress and burnout^7,8^. Additionally, psychological distress among students has been claimed to have a negative impact on academic performance^9^, to increase thought of dropping out medical school^10^, to lead to academic dishonesty including problems such as plagiarism, cheating, pseudoscience and falsification^11^, and to alcohol and substance addiction^12,13^. Medical students suffering from psychological distress have shown cynicism^14^, reluctance to care for the chronically ill patients^15^, and more generally lack of empathy^16^.

To make things worse, the recent Covid-19 pandemic has placed an additional burden on students around the world^17^. Among the many sources of distress, the most relevant have been the loss of peer contact and social connectivity, financial pressures due to events such as the loss of part-time jobs. All these situations predicted to exacerbate psychological discomfort and to disrupt medical students' everyday lives and studies^18,19^. Concerns about their own and their families' health and well-being, as well as the prospect of being fast-tracked to the frontline or sent to other locations of the health service, may have added to their stress levels during the pandemic time^20^.

Such a situation is obviously not desirable since, other than putting medical students under a heavy mental distress, it gives space to numerous risk factors that can cause the development of later clinical mental conditions^21^.

Aware of this situation, medical students are using different coping strategies to reduce distress. While taking drugs and substances, such as tranquilizers, stimulants, and alcohol, overthinking and rumination have negative or side effects, taking social support such as seeking help from friends or family and physical activity have positive effects to reduce distress^22^.

Mindfulness-Based Intervention (MBI) is an effective complement to these strategies. Defining mindfulness is not an easy task, however, a generally accepted and synthetic definition of this concept can be found in the work of Jon Kabat-Zinn, where it is formulated as “*the awareness that emerges through paying attention on purpose, in the present moment, and nonjudgmentally, to the unfolding of experience moment by moment*”^23^. Mindfulness is a state of pre-reflexive consciousness that can be put in contrast with other modalities in which the person is constantly engaged in overthinking, obsessing about the past, fantasizing and worrying about the future or engaged in compulsive or automatic behavior without acknowledging it^24^.

The benefits of such a state of being is well-known in literature. Empirical research shows that mindfulness-based therapies can reduce stress and anxiety, as well as psychological distress, chronic pain, and depression, and improve overall quality of life^25–28^.

For medical students MBIs for reducing stress include Mindfulness-Based Stress-Reduction (MBSR) or meditation techniques, self-hypnosis, and pass/fail grading^29^, this latter generates less pressure among students because they are not actively competing with their peers or worrying about letters or numbers.

MBIs are getting popular in medical education to such an extent that medical schools around the world are beginning to acknowledge mindfulness as a helpful practice for their students, residents, and staff^30^. In addition, these programs explore how to incorporate mindfulness into therapeutic practice^31,32^ with the aim of improving physician self-care, self-awareness, and empathy, with the goal of mutually enhancing physician well-being and patient care quality. A recent meta-analysis showed the efficacy of MBIs in reducing and preventing stress and burnouts on medical students^33^. Researches on the general population reported a positive correlation of mindfulness with high levels of optimism, pleasant effect, perception of autonomy^34^ and empathy^35^. Further study demonstrated that individuals with higher levels of trait mindfulness have lower levels of difficulties in emotion regulation^36^. A study among nursing students having stressful educational programs showed that trait mindfulness was negatively related to psychological distress^37^. Another studied demonstrated the beneficial role of mindfulness facets in mitigating negative consequences of trait anxiety on medical student well-being during high-pressure periods^38^.

These data take on further importance considering the result of the medical service depends not only on the doctor's ability to diagnose and treat a disease, but also on the ability to establish a positive relationship with the patient.

Additionally, mindfulness training, directed to build a metacognitive standpoint toward the thinking process, could reduce the great number of misdiagnoses attributed to cognitive biases such as anchoring mistakes or attribution error^39^.

Hence to develop and apply easily accessible and low-cost strategies to diminish the levels of stress of medical students is crucial for their academic performance and professional development.

The aim of the present study was to assess the efficacy of a comprehensive MBI, entirely administered online, consisting of a mindfulness-based practice, called Integral Meditation (IM), dietary advice, and brief yoga sessions, in two cohorts of Italian medical students randomized to intervention or control group. Our primary hypothesis was that participants in the intervention group would report a decrease of perceived stress and state anxiety and an increase of mental well-being. Our secondary hypothesis was that participants in the intervention group would report higher scores on different measures of positive affect, resilience, emotion regulation and attentional control. The investigated outcomes were measured using nine self-report questionnaires administered online to both groups before and after the intervention. For the second cohort only, 3 months follow-up data was also collected and analyzed.

## Results

**Figure 1:**
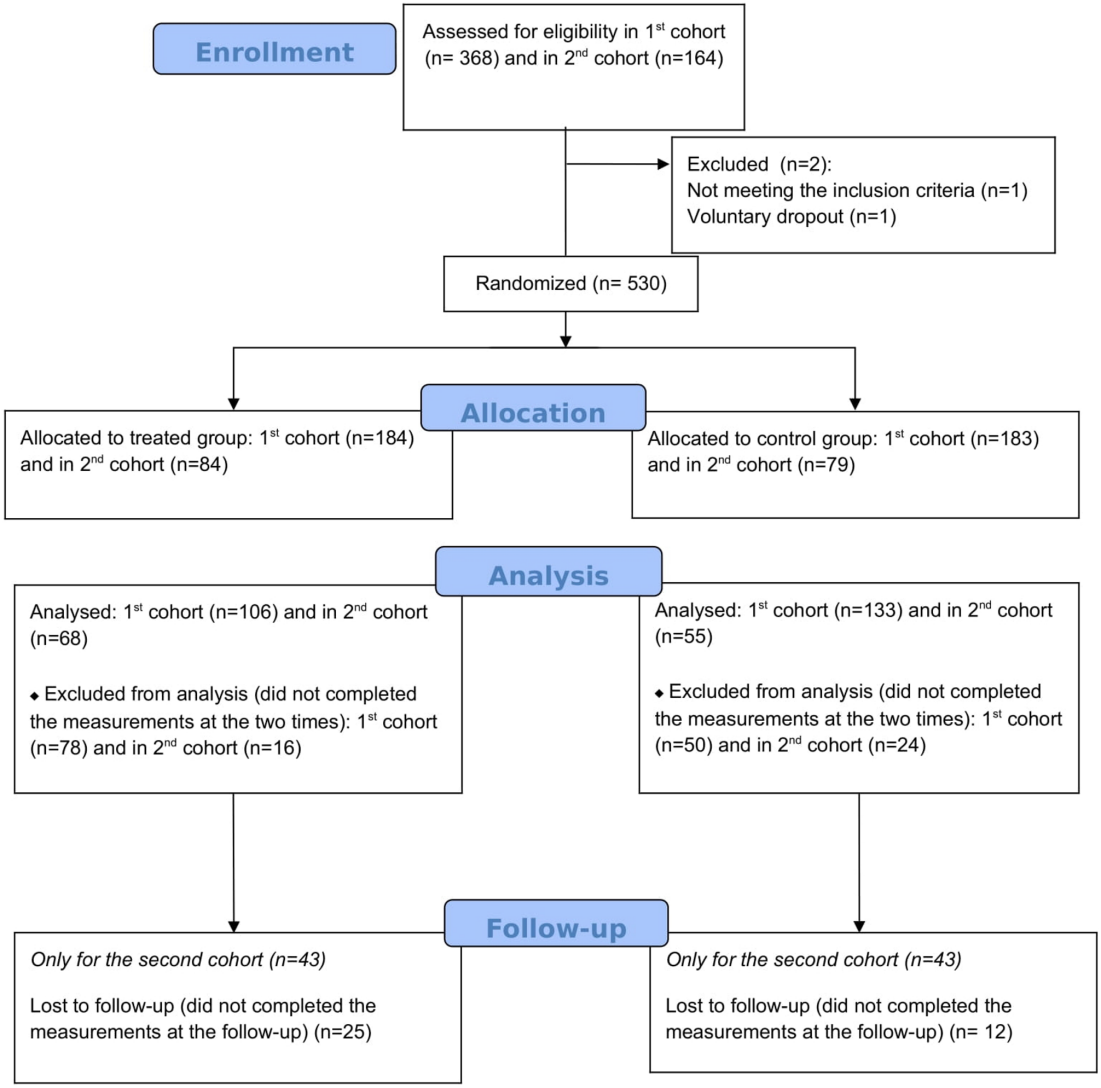
Participant flow diagram.

Considering the two recruitment calls, a total of 532 participants were enrolled for the study; among these, 530 were eligible for the analysis (367 in cohort 1 and 163 in cohort 2) and randomly allocated to one of the two groups. It should be noted that 128 participants (50 controls and 78 treated) in the first cohort and 40 participants (24 controls and 16 treated) in the second cohort dropped out after the first few IM classes, or even before the start of the intervention, for logistical and/or personal reasons. They were excluded from the analysis since they did not complete the administered questionnaires both at baseline and, even more, at the end of the study (see Fig. 1). Out of 362 analyzed subjects, age ranged 19–52 (interquartile range = 21–24), 239 belonged to the first cohort (106 treated and 133 controls) and 123 (68 treated and 55 controls) to the second cohort.

Each participant in both cohorts and allocation groups filled in the administered questionnaires at two time point: before the intervention (t0) and after the intervention (t1). For 86 subjects (43 treated and 43 controls) in the second cohort we also collected and analyzed 3-months follow-up questionnaires data (t2). Students in the two cohorts attended the MBI classes in two different time periods: the first cohort in March–April and the second in May–June.

A post-hoc sensitivity analysis was performed to assess the minimum detectable effect size with our collected sample (n = 362). Fixing α = 0.05 two-sided and a 90% of power in detecting differences between groups, our sample allow to detect an effect size d = 0.30.

The baseline characteristics of the sample in the two cohorts are reported globally and separately in the two groups within each cohort in Supplementary Table 1 and in Supplementary Table 2 respectively. As reported in Supplementary Table 1 statistically significant baseline differences between the two cohorts were observed for university (p < 0.0001), academic course language (p < 0.0001), academic course year (p < 0.0001), housing situation (p < 0.0001), work (p = 0.009), sport activities (p = 0.04), previous meditation experience (p = 0.005), competition (p = 0.04) and feeling stressed due to COVID-19 situation (p = 0.007) variables. On the contrary, no statistically significant differences were observed between the two groups within each cohort (Supplementary Table 2) except for cultural/sport association membership (p = 0.03).

In Table 1 mean and Standard Deviation (SD) for each questionnaire and subscale, both at t0 and t1, and in the second cohort also at t2, are reported in the control and in the treated group for the whole sample and separately for the two cohorts. Moreover, p-values for the baseline differences between treated and controls were also calculated for each investigated endpoint and reported in the table. In Supplementary Table 3 the internal consistency for each questionnaire calculated at both t0 and t1, separately for each cohort and considering the whole sample are reported. Table 2 reports for each questionnaire and its subscales, β coefficients of time*treatment interaction, its corresponding 95% Confidence Interval (CI), and the p-value for the analysis of the whole sample. For all the investigated outcomes, except for STAI-X1, a statistically significant time*group interaction, after multiple testing correction, was observed, i.e., the change in score over time differed between the two groups. Specifically, our intervention was effective in: reducing perceived stress, as measured by PSS (β = − 2.57 [95%CI = − 4.02; − 1.12], p = 0.004) improving mental well-being, as measured by WEMWBS (β = 2.82 [95%CI = 1.02; 4.63], p = 0.008), improving emotional regulation, as measured by the whole scale of DERS (β = − 8.24 [95%CI = − 12.98; − 3.51], p = 0.004) as well as its subscales: *lack of acceptance* (β = − 1.04 [95%CI = − 2.76; − 0.05], p = 0.04), *difficulties in distraction* (β = − 1.07 [95%CI = − 2.04; − 0.11], p = 0.04), *lack of trust* (β = − 2.10 [95%CI = − 3.48; − 0.72], p = 0.008), *lack of control* (β = − 1.31 [95%CI = − 2.49; − 0.13], p = 0.04), *difficulties in recognition* (β = − 1.55 [95%CI = − 2.62; − 0.48], p = 0.01), and *reduced self-awareness* (β = − 0.80 [95%CI = − 1.39; − 0.22], p = 0.01). A statistically significant effect was also observed in ameliorating: resilience, as measured by RS-14 (β = 3.79 [95%CI = 1.32; 6.26], p = 0.008), the tendency to wander with the mind, as measured by MW-S (β = − 0.7 [95%CI = − 0.99; − 0.39], p = 0.0001), the ability to maintain attention (as measured by AC-S (β = − 0.23[95%CI = − 0.44; − 0.02], p = 0.04) and AC-D (β = − 0.19[95%CI = − 0.36; − 0.01], p = 0.04)) and the overall distress, as measured by the Positive PANAS (β = 1.84 [95%CI = 0.45; 3.23], p = 0.02), and the negative PANAS (β = − 2.04 [95%CI = − 3.69; − 0.40], p = 0.02).

**Table 1:** Mean and standard deviation (SD) for each questionnaire and subscale in the two groups (controls and treated) at t0, t1 were calculated for the whole sample and separately for the two cohorts.

Questionnaire	Mean (SD) Controls t0	Mean (SD) Treated t0	*p*^a^	Mean (SD) Controls t1	Mean (SD) Treated t1	Mean (SD) Controls t2	Mean (SD) Treated t2
STAI-X1							
All sample	48.17 (12.63)	45.58 (12.43)	0.04	49.00 (11.51)	44.22 (10.92)	–	–
Cohort 1	48.16 (12.5)	45.32 (12.1)	0.05	48.2 (11.36)	43.44 (11.33)	–	–
Cohort 2	48.2 (13.1)	45.98 (13.02)	0.35	50.94 (11.72)	45.44 (10.18)	46.07 (11.97)	40.51 (10.56)
STAI-X2							
All sample	52.70 (10.47)	51.25 (10.92)	0.15	–	–	–	–
Cohort 1	52.48 (9.95)	51.41(10.9)	0.43	–	–	–	–
Cohort 2	53.21(11.7)	51.00(11.04)	0.29	–	–	–	–
PSS							
All sample	23.96 (6.12)	22.58 (6.40)	0.04	22.49 (5.60)	18.63 (6.02)	–	–
Cohort 1	24.30 (5.95)	23.07 (6.41)	0.13	22.44 (5.5)	18.21 (6.34)	–	–
Cohort 2	23.12 (6.49)	21.80 (6.33)	0.26	22.61 (5.87)	19.26 (5.46)	22.41 (7.03)	17.35 (6.51)
WEMWBS							
All sample	42.97 (7.94)	43.90 (8.30)	0.28	43.25 (7.56)	47.04 (7.65)	–	–
Cohort 1	42.69 (7.79)	43.31 (8.38)	0.56	43.40 (7.2)	47.56 (7.93)	–	–
Cohort 2	43.63 (8.29)	44.82 (8.14)	0.43	42.87 (8.4)	46.22 (7.17)	43.25 (7.96)	48.58 (8.39)
MW-S							
All sample	4.97 (1.34)	4.90 (1.39)	0.65	4.90 (1.29)	4.16 (1.39)		
Cohort 1	5.02 (1.36)	4.94 (1.42)	0.69	5.03 (1.27)	4.05 (1.36)	–	–
Cohort 2	4.83 (1.31)	4.84 (1.36)	0.90	4.59 (1.28)	4.34 (1.44)	4.58 (1.2)	4.02 (1.47)
PANAS* Positive*							
All sample	29.45 (6.22)	31.33 (7.28)	0.01	29.30 (7.02)	32.99 (6.52)		
Cohort 1	29.49 (5.95)	31.18 (7.31)	0.06	29.12 (6.66)	33.34 (6.69)	–	–
Cohort 2	29.34 (6.89)	31.54 (7.28)	0.09	29.7 (7.86)	32.42 (6.24)	29.93 (7.64)	32.86 (6.58)
PANAS *Negative*							
All sample	30.68 (7.89)	29.06 (7.70)	0.04	29.38 (7.89)	25.77 (7.43)		
Cohort 1	30.60 (8.06)	29.11 (7.65)	0.10	29.45 (8.09)	25.55 (8.08)	–	–
Cohort 2	30.87 (7.54)	28.98 (7.83)	0.18	29.20 (7.45)	26.1 (6.31)	29.02 (7.07)	23.86 (7.16)
DERS *All items*							
All sample	97.49 (22.41)	95.33 (24.07)	0.38	97.18 (22.31)	86.67 (21.83)		
Cohort 1	98.00 (22.49)	96.83 (23.61)	0.70	98.00 (22.51)	88.04 (22.59)	–	–
Cohort 2	96.23 (25.00)	92.98 (21.95)	0.44	95.18 (21.85)	84.51 (20.57)	95.32 (21.95)	79.72 (18.89)
DERS *Lack of acceptance*							
All sample	17.50 (6.70)	16.84 (7.12)	0.34	17.31 (6.80)	15.13 (6.43)		
Cohort 1	17.9 (6.67)	17.38 (7.1)	0.56	17.78 (6.81)	15.84 (6.5)	–	–
Cohort 2	16.52 (6.7)	15.98 (7.12)	0.61	16.14 (6.7)	14.01 (6.2)	16.48 (6.38)	12.7 (5.19)
DERS *Difficulties in distraction*							
All sample	19.70 (4.07)	18.79 (4.46)	0.06	18.99 (4.14)	16.99 (4.28)		
Cohort 1	19.75 (4.07)	18.74 (4.44)	0.09	19.42 (3.89)	16.84 (4.30)	–	–
Cohort 2	19.54 (4.09)	18.85(4.52)	0.48	17.92 (4.54)	17.200 (4.26)	18.7 (4.56)	15.67 (3.9)
DERS *Lack of trust*							
All sample	23.80 (6.62)	22.78 (7.35)	0.18	23.69 (6.22)	20.50 (6.29)		
Cohort 1	23.97 (6.45)	23.00 (7.49)	0.23	23.78 (6.44)	20.67 (6.59)	–	–
Cohort 2	23.38 (7.04)	22.42 (7.16)	0.46	23.45 (5.68)	20.22 (5.81)	24.02 (6.09)	19.65 (5.94)
DERS *Lack of control*							
All sample	15.05 (5.84)	15.65 (6.11)	0.47	16.04 (5.68)	14.33 (5.32)		
Cohort 1	23.78 (5.68)	20.67 (6.2)	0.59	16.15 (5.75)	14.4 (5.38)	–	–
Cohort 2	23.45 (6.27)	20.22 (5.98)	0.64	15.74 (5.54)	14.22 (5.25)	16.04 (5.35)	12.86 (4.8)
DERS *Difficulties in recognition*							
All sample	13.72 (4.67)	14.33 (4.90)	0.23	13.94 (4.29)	13.06 (4.51)		
Cohort 1	13.6 (4.47)	14.79 (4.42)	0.06	13.84 (4.47)	13.50 (4.42)	–	–
Cohort 2	13.98 (3.86)	13.60 (4.59)	0.68	14.16 (3.86)	12.35 (4.59)	13.34 (3.99)	12 (4.37)
DERS *Reduced self-awareness*							
All sample	6.72 (2.88)	6.95 (3.12)	0.60	7.21 (3.09)	6.65 (2.89)		
Cohort 1	6.72 (2.87)	7.00 (3.09)	0.44	6.99 (3.01)	6.75 (2.74)	–	–
Cohort 2	6.7 (2.93)	6.75 (3.18)	0.99	7.74 (3.22)	6.5 (3.12)	6.73 (2.8)	6.84 (3.15)
RS-14							
All sample	65.66 (13.01)	65.56 (13.57)	0.37	64.32 (13.39)	69.00 (13.34)		
Cohort 1	65.53 (12.8)	64.79 (14.21)	0.91	64.36 (13.54)	68.66 (13.79)	–	–
Cohort 2	65.98 (13.6)	69.32 (12.09)	0.17	64.20 (13.12)	69.52 (12.67)	61.66 (13.34)	71.44 (14.17)
AC-S							
All sample	2.98 (1.06)	2.81 (1.15)	0.12	3.11 (0.96)	2.73 (1.08)		
Cohort 1	2.97 (1.03)	2.90 (1.19)	0.53	3.12 (0.97)	2.75 (1.12)	–	–
Cohort 2	3.00 (1.13)	2.66 (1.07)	0.10	3.13 (0.93)	2.68 (1.03)	3.05 (1.01)	2.49 (0.90)
AC-D							
All sample	3.62 (0.91)	3.53 (0.89)	0.37	3.57 (0.92)	3.30 (0.96)		
Cohort 1	3.55 (0.94)	3.58 (0.91)	0.81	3.55 (0.96)	3.25 (1.03)	–	–
Cohort 2	3.77 (0.79)	3.46 (0.86)	0.04	3.61 (0.83)	3.38 (0.84)	3.54 (0.91)	2.99 (0.92)

For the 2nd cohort only, mean and SD at follow-up (t2) were also reported. The table also reports the p-value (*p*) for the baseline (t0) differences between treated and controls.

^a^p-value for the baseline (t0) differences between treated and controls calculated for the whole sample and separately for each cohort.

**Table 2:** Linear mixed model results considering the whole sample (n = 356 students).

All sample (cohort 1 + cohort 2) (n = 356 students)			
Questionnaire	β time*group [95%CI]	Adjusted p-value	Effect size (Hedges’s)
STAIX-1	− 2.23 [− 4.94; 0.49]	0.11	− 0.16 (negligible)
PSS	− 2.57 [− 4.02; − 1.12]	**0.004**	**− 0.36 (small)**
WEMWBS	2.82 [1.02; 4.63]	**0.008**	**0.33 (small)**
MW-S	− 0.70 [− 0.99; − 0.39]	**0.0001**	**− 0.47 (small)**
PANAS *Positive*	1.84 [0.45; 3.23]	**0.02**	**0.27 (small)**
PANAS *Negative*	− 2.04 [− 3.69; − 0.40]	**0.02**	**− 0.25 (small)**
DERS *All items*	− 8.24 [− 12.98; − 3.51]	**0.004**	**− 0.37 (small)**
DERS *Lack of acceptance*	− 1.04 [− 2.76; − 0.05]	**0.04**	**− 0.23 (small)**
DERS *Difficulties in distraction*	− 1.07 [− 2.04; − 0.11]	**0.04**	**− 0.24 (small)**
DERS *Lack of trust*	− 2.10 [− 3.48; − 0.72]	**0.008**	**− 0.33 (small)**
DERS *Lack of control*	− 1.31 [− 2.49; − 0.13]	**0.04**	**− 0.23 (small)**
DERS *Difficulties in recognition*	− 1.55 [− 2.62; − 0.48]	**0.01**	**− 0.29 (small)**
DERS *Reduced self-awareness*	− 0.80 [− 1.39; − 0.22]	**0.01**	**− 0.28 (small)**
RS-14	3.79 [1.32; 6.26]	**0.008**	**0.32 (small)**
AC-S	− 0.23 [− 0.44; − 0.02]	**0.04**	**− 0.22 (small)**
AC-D	− 0.19 [− 0.36; − 0.01]	**0.04**	**− 0.22 (small)**

For each questionnaire and subscale β coefficient of time*group interaction with its 95% CI, and p-value are reported together with the Hedges’s effect size.

All the models were adjusted for age, sex, previous meditation experience, the baseline value of STAI-X2, the cohort variables and the propensity score (PS) comprising 8 possible additional confounding variables (i.e., university, course language, academic course year, housing situation, work, sport activities, feeling stressed due to covid situation, feel competition among students).

Significant values are in bold.

An additional analysis considering separately the two cohorts was also performed at the light of the statistically significant differences between them and results are reported in Table 3. As for the cohort 1, a statistically significant time*group interaction, after multiple testing correction, was observed for almost all the investigated outcomes, In particular a statistically significant decrease was found for PSS (β = − 3.00 [95%CI = − 4.54; − 1.46], p = 0.0006), MW-S (β = − 0.91 [95%CI = − 1.21; − 0.61], p < 0.0001), PANAS negative (β = − 2.41 [95%CI = − 4.13; − 0.68], p = 0.009), the whole scale of DERS (β = − 8.78 [95%CI = − 13.36; − 4.21], p = 0.004) and all its subscales: *difficulties in distraction* (β = − 1.56 [95%CI = − 2.55; − 0.58], p = 0.004), *lack of trust* (β = − 2.13 [95%CI = − 3.48; − 0.79], p = 004), *lack of control* (β = − 1.55 [95%CI = − 2.74; − 0.37], p = 0.01) and *difficulties in recognition* (β = − 1.52 [95%CI = − 2.63; − 0.42], p = 0.009), AC-S (β = − 0.29 [− 0.5; − 0.09], p = 0.009) and AC-D (β = − 0.33 [95%CI = − 0.5; − 0.16], p = 0.0006). A statistically significant increase was instead observed for both the WEMWBS (β = 3.55 [95%CI = − 1.7; 5.39], p = 0.0006), Positive PANAS (β = 2.53 [95%CI = 1.13; 3.92], p = 0.001), and the RS-14 (β = 5.04 [95%CI = 2.69; 7.40], p = 0.0003). As for the cohort 2, which attended the training in the period May–June, no statistically significant effect of intervention, after multiple testing corrections, was detected for the investigated endpoints. For this cohort we also collected follow-up data 3 months after the last IM sessions. Despite the lack of statistically significant results after correcting for multiple testing, the follow-up data analysis revealed, as reported in Table 4**,** a suggestive significant decrease in PSS (β = − 3.55 [95%CI = − 6.77; − 0.34], p = 0.03), and DERS total score (β = − 11.11 [95%CI = − 22.12; − 0.12], p = 0.049) and in two of its subscales, *lack of acceptance* (β = − 2.97 [95%CI = − 4.48; − 0.12], p = 0.049) and *difficulties in distraction* (β = − 2.3 [95%CI = − 4.19; − 0.04], p = 0.04), as well as an increase for RS-14 (β = 5.52 [95%CI = 0.04; 11], p = 0.05).

**Table 3:** Linear mixed model results separately for each cohort. For each questionnaire and subscale β coefficient of time*group interaction with its 95% CI, and p-value are reported.

Questionnaire	Cohort 1		Cohort 2	
	β time*group [95%CI]	Adjusted p-value	β time*group [95%CI]	Adjusted p-value
STAIX-1	− 1.92 [− 4.81; 0.98]	0.19	− 3.29 [− 8.72; 2.14]	0.55
PSS	− 3.00 [− 4.54; − 1.46]	**0.0006**	− 2.04 [− 4.78; 0.71]	0.55
WEMWBS	3.55 [1.7; 5.39]	**0.0006**	2.16 [− 1.51; 5.83]	0.55
MW-S	− 0.91 [− 1.21; − 0.61]	** < 0.0001**	− 0.26 [− 0.91; 0.39]	0.6
PANAS *Positive*	2.53 [1.13; 3.92]	**0.001**	0.52 [− 2.57; 3.6]	0.79
PANAS *Negative*	− 2.41 [− 4.13; − 0.68]	**0.009**	− 1.21 [− 4.56; 2.14]	0.65
DERS *All items*	− 8.78 [− 13.36; − 4.21]	**0.004**	− 7.42 [− 17.35; 2.51]	0.55
DERS *Lack of acceptance*	− 1.42 [− 2.89; 0.04]	0.06	− 1.59 [− 4.47; 1.29]	0.55
DERS *Difficulties in distraction*	− 1.56 [− 2.55; − 0.58]	**0.004**	− 0.29 [− 2.09; 2.03]	0.98
DERS *Lack of trust*	− 2.13 [− 3.48; − 0.79]	**0.004**	− 2.28 [− 5.14; 0.58]	0.55
DERS *Lack of control*	− 1.55 [− 2.74; − 0.37]	**0.01**	− 0.8 [− 3.40; 1.79]	0.65
DERS *Difficulties in recognition*	− 1.52 [− 2.63; − 0.42]	**0.009**	− 1.43 [− 3.73; 0.87]	0.55
DERS Reduced self-awareness	− 0.58 [− 1.18; 0.009]	0.06	− 1.29 [− 2.56; − 0.008]	0.55
RS-14	5.04 [2.69; 7.40]	**0.0003**	1.99 [− 3.65; 7.63]	0.65
AC-S	− 0.29 [− 0.5; − 0.09]	**0.009**	− 0.1 [− 0.57; 37]	0.79
AC-D	− 0.33 [− 0.5; − 0.16]	**0.0006**	0.07 [− 0.31; 0.46]	0.79

All the models were adjusted for age, sex, previous meditation experience, academic course year and the baseline value of STAI-X2.

Significant values are in bold.

**Table 4:** Linear mixed model results for the follow-up analysis performed on the 2nd cohort only.

Questionnaire	β time*group [95%CI]	Unadjusted p-value	Adjusted p-value
PSS	− 3.55 [− 6.77; − 0.34]	0.03	0.16
WEMWBS	3.21 [− 0.78; 7.19]	0.11	0.20
MW-S	− 0.59 [− 1.28; 0.11]	0.10	0.20
PANAS *Positive*	0.12 [− 3.11; 3.34]	0.94	0.94
PANAS *Negative*	− 3.39 [− 7.06; 0.7]	0.07	0.16
DERS *All items*	− 11.11 [− 22.12; − 0.11]	0.05	0.16
DERS *Lack of acceptance*	− 2.97 [− 4.48; − 0.12]	0.05	0.16
DERS *Difficulties in distraction*	− 2.3 [− 4.19; − 0.04]	0.04	0.16
DERS *Lack of trust*	− 3[− 6.24; 0.24]	0.07	0.16
DERS *Lack of control*	− 1.44 [− 4.2; 1.32]	0.30	0.41
DERS *Difficulties in recognition*	− 1.28 [− 3.58; 1.02]	0.27	0.41
DERS *Reduced self-awareness*	− 0.12 [− 1.35; 1.12]	0.85	0.91
RS-14	5.49 [0.02; 10.95]	0.05	0.16
STAI X-1	− 3.33 [− 9.59; 2.93]	0.3	0.41
AC-S	− 0.04 [− 0.15; 0.08]	0.49	0.61
AC-D	− 0.07 [− 0.46; 0.32]	0.73	0.83

All the models were adjusted for age, sex, previous meditation experience, academic course year and the baseline value of STAI-X2.

For each questionnaire and subscale β coefficient of time (t2 vs t0)*group interaction with its 95% CI, and p-value are reported.

Lastly, we conducted a multiple mediation post-hoc analysis to investigate the direct effect of our intervention on well-being, assessed through WEMWBS, and the indirect effect mediated by changes in attentional control, measure via AC-S and AC-D, and emotional regulation, measured via DERS. Results showed a statistically significant direct effect of the proposed intervention β = 1.56 (0.11; 3.00) p = 0.04 and an indirect effect through DERS β = − 1.73 (0.70; 2.76), p = 0.001.

### Qualitative comparison of average scores for the medical students with general population

Students from both the cohorts at t0 reported higher mean scores in most of the evaluated outcomes with respect to the values found in the literature. They showed higher values of perceived stress compared to a mean score of 15.9 ± 6.3 reported by the authors of the Italian PSS for the subjects aged ≤ 30 (N = 80)^40^, and a mean score above 40 that has been indicated as a cutoff to detect clinically significative symptoms^41^. Our sample also reported a mean score of MW-S higher compared to 3.16 ± 0.79, which the authors of the original version of MW-S found over a population of Canadian students (N = 192)^42^, a higher mean score in the negative affect subscale of PANAS compared to mean score of 16 ± 6.2 in the negative affect subscale found in a sample of N = 600 participants, with a mean age of 27.9 ± 9.78^43^. As regard to emotional regulation, our sample also had a mean score higher than the score reported by the authors of the Italian DERS, i.e., 61.38 ± 15.37 among a sample of N = 190 participants with a mean age of 30.8 ± 9.7^44^. High perceived stress, anxiety, mind wandering, negative effects, and difficulties in emotional regulation depict a dauting situation of the medical students in Italian Universities. Moreover, the participants showed a lower score in resilience compared to a mean score of 76.13 ± 10.48 found in Callegari et al.^45^ among a sample of participants (N = 150) composed by 85% of people aged between 18 and 35 years old. The presence of high anxiety, stress and negative emotion might be caused or exacerbated by the issues related with attending a medical school, and the presence of high difficulties in emotional regulation and the low resilience might indicate scarce individual resources to face these difficulties.

## Discussions

Medical students have a significantly higher stress level than general population^1^. Thus, the development and application of easily-accessible and low-cost intervention aimed at reducing stress and improving the well-being of medical students are getting considerable interest. Reducing stress and increasing well-being will in turn also improve their academic performance and the relationship with the patient with consequent notable advantages in clinical practice.

The present study was performed on a large sample of medical students from Italian different universities recruited in two different cohorts and randomized to treated or control group. The treated group underwent a comprehensive MBI whose core element consisted of 10 online IM classes, given twice a week via Zoom videoconferencing platform^46^, lasting approximately 35 min each. The IM classes were preceded by 10 min of brief yoga exercises. As part of the comprehensive intervention, students have also received dietary advice.

The main analysis was performed on the whole recruited sample of 362 medical students by also adjusting the model for all the measured confounding variables statistically significant different between the two cohorts and which were included in a Propensity Score (PS).

Our intervention showed a statistically significant effect, except for STAI-X1, in all the investigated endpoint, confirming our hypothesis of the beneficial effect of our MBI. Specifically, the intervention emerged as effective in: (*i*) reducing perceived stress, (*ii*) improving mental well-being, (*iii*) improving emotional regulation, and (*iv*) resilience, (*v*) reducing the tendency to wondering with the mind, (*vi*) improving the ability to maintain attention and (*vii*) reducing the overall distress.

Given the presence of statistically significant differences between the two cohorts in many informative background variables collected at baseline, indicating that the samples were quite different, although both belonging to the population of medical students, we further performed a secondary analysis separately on the two cohorts. The differences between the two cohorts, also considering those unmeasured, could have affected the effectiveness of our MBI. Furthermore, students in the two cohorts were recruited and attended the intervention in two different time periods: the first cohort in March–April and the second in May–June, this latter close to the exam session. The pandemic situation, whose restrictive measures have been greatly relaxed over the months, may have played an important role with a consequent benefit for the social lives of the students. Being these factors numerous and difficult to formalize, since many of them were probably not observed and even unknown, we could not have included all the possible confounding variables in the statistical models or in the creation of the PS in our main analysis. In addition, our administered background questionnaire, albeit enriched with questions related to different characteristics (from personal data to lifestyle), is not exhaustive of all possible variables describing the single subject, and hence not able to capture all the sources of variation between the two cohorts.

This secondary analysis showed that the students belonging to the first cohort were positively affected by our intervention, in almost all areas of experimentation, confirming our hypothesis of the beneficial effect of our MBI. Specifically, compared to the control group, the intervention group had reported lower scores on measures of perceived stress, anxiety, and higher scores on different measures of positive affect, resilience, mental well-being, and emotion regulation. On the contrary, students from the second cohort seem not to have benefited from our MBI and this could depend by unknown differences between the two sample, other than the confounding variables included in the model.

We also performed a multiple mediation post-hoc analysis to investigate if changes in emotional regulation, attentional control and attentional shift could mediate the effect of our intervention on increasing participants’ psychological well-being. We found a significant indirect effect of our intervention on psychological well-being mediated by reduced difficulties in emotional regulation, while no indirect effect for attentional control and attentional shift was found. These results support the idea that MBIs could improve medical students’ well-being through different paths: increasing the well-being itself but also improving other aspects that lead to an increased well-being, such as difficulties in emotional regulation. Our results are in line with what is reported by Tang et al.^47^ “*enhanced emotion regulation has been suggested to underlie many of the beneficial effects of mindfulness meditation*”.

In this study we observed a gender difference, more females than males, in reaching out to the program as also found in literature^48^. Even though overall both females and males report that meditation is useful to reduce stress and to improve well-being for different reasons, females believe it more than males^49^. Therefore, females may had been more motivated to participate in our intervention as indicated by the different portion of the two genders in our sample.

The limitations of our study were: (i) given the voluntary nature of the participation, we were unable to extrapolate the benefits of the program to the general population. So, the inference can only be extended to those students who decide to attend meditation classes if offered. It should be emphasized that to benefit from a program like MBI, individuals must be really determined to meditate, therefore participation must necessarily be on a voluntary basis; and (ii) the solely use of self-report measures: the reliability of self-report measures may have influenced our findings^50^ and the length of the surveys may have caused respondent fatigue^51^.

Despite the reported limitation the strength of our papers resides in the large sample recruited and in the randomized assignment to the treated and control group. Furthermore, as to our knowledge, this is the first study aimed at investigating the effect of a MBI, as easily accessible and low-cost intervention, on a broad range of psychological indicators in medical students and during the Covid-19 pandemic time.

In addition, the high percentages of students with irregular sleep (over 30%) and with a recent psychological consultation (approximately 25%) that we found in this study highlight the need for preventive intervention addressing medical students’ health, and mindfulness meditation seems to be an optimal candidate to cover such a role.

In conclusion, taken all together, the results obtained show a general improvement of well-being at the end of the treatment program therefore confirming the positive effects of practices of mindfulness^25–28^. Our results show that a short mindfulness practice has an immediate impact on emotional functioning and on different psychological indicators of well-being in medical students. Well-being is one of the factors that may be indirectly related to clinical misdiagnosis or errors^52^. Therefore, improving the well-being of medical students may reduce attribution errors and misdiagnosis in future clinical practice. Improvements in emotional regulation, in resilience and in the dimension of positive affect, can lead to the achievement of transversal skills useful not only when studying but also in the future medical profession, as well as represent themselves personological traits of personal reference. For example, the improvements in the tendency to wander with the mind, and in the ability to maintain attention appears important for the purpose of regular attendance of the course of study and the quality of learning, considering the fair percentage of students (approximately 25%) who in our sample reported being not in compliance with their exams.

For all these reasons we are confident in concluding the MBI it is a practice that it is worthwhile adopting also to improve students’ well-being and quality of life.

## Materials and methods

### Participants, recruitment process and allocation procedure

The study was promoted by the Universities of Pavia and Brescia, but the participation was extended to every Italian university. Participants were recruited via digital (social networks and mailing) and non-digital means (word of mouth) in two different recruitment calls (spring and summer calls) therefore representing two different cohorts. Participation was on a voluntary basis; this implies that most students who joined the study were probably inclined and interested towards mindfulness and/or meditation. To promote the participation of many people we offered prizes to a small group of participants drawn by lot.

The inclusion criteria were: (*i*) not suffering at the time of recruitment from severe anxiety or depression, severe mental illness (e.g., hypomania or psychotic episode), or any other serious mental or physical health problem; (*ii*) to be 18 or older; (*iii*) to have a digital device and an Internet connection to complete the assessment and to attend the classes; (*iv*) to understand Italian language. These criteria were clearly stated during the recruitment call and were further assessed by asking the participants of both groups’ straight questions on these issues. Each eligible participant received a leaflet outlining the study's aims, design, and timeline, as well as the informed consent and the privacy policy. All the subjects had to read and agree with the privacy policy and to sign the informed consent sent by email. The first cohort started the intervention the 15th of March 2021 and completed it by the 21st of April, while the second cohort started the intervention by 10th May 2021 and completed it by 16th June 2021. In each cohort eligible participants were randomly allocated with 1:1 ratio to intervention or control group by using the R functions split and set.seed to ensure the reproducibility of the results. 

Due to the nature of the intervention, participants were aware of their group assignment, were invited to complete all the online questionnaires, and as for the subjects assigned to the intervention group, they were also invited to attend the training sessions on the given dates. In both cohorts, the individuals assigned to the passive control group did not attend any training session and were only asked to complete the same questionnaires as the treatment group. For ethical reasons, to the control group was offered the same intervention at a subsequent time.

### Intervention

The comprehensive MBI, entirely administer online via Zoom platform^46^, included: (i) 10 IM classes lasting approximately 35 min each, given twice a week on Monday and Wednesday; (ii) 10 min of yoga exercises focused on breathing and posture before each IM class; and (iii) dietary advice. Regarding this latter, the participants received via email a document which entailed non-mandatory dietary suggestions to promote healthy nutrition, sleep, and stress-relief. An online lecture held by the nutritionist with a question-and-answer format was also organized. The degree to which to follow this advice was left to the discretion of each participant.

The core of our intervention was represented by the IM training, a MBI intervention well accepted by both novice and experienced meditators, that has proven efficacy in the non-clinical general population as reported in previous studies^53–57^. IM simultaneously uses breathing, focusing attention, release of physical tensions, thoughts and feeling sensations through internal senses and imagery, allowing a quick relaxation and more deeply a physical, energetic, and spiritual well-being.

Compared to our first works^53–55^, here IM was entirely administered online, reducing the classes from 12 one-a-week and lasting 60 min each to 10 twice-a-week and lasting 35 min each, to guarantee a better adherence and participation.

To not influence the results, participants in the control group were asked not to practice meditation during the period of the study.

Each cohort had its own calendar organized into:

- *Meeting 1:* Introduction to the course, brief explanation of mindfulness (e.g., breath, posture, etc.) and nutritionist intervention illustrating the non-mandatory dietary suggestions.
- *Meetings 2–6:* 10 min Yoga sessions plus 35 min IM sessions.
- *Meeting 7:* Lecture held by a nutritionist with a question-and-answer format.
- *Meetings 8–12:* 10 min Yoga sessions plus 35 min IM sessions.

An expert meditator trainer developed and administered the training.

### Measures

Each participant in both groups and cohorts at the different time points, completed online via Google Forms the Italian validated version of nine questionnaires:

i. Perceived Stress Scale (PSS)^40,58^ for measuring the perception of stress;
ii. Warwick-Edinburgh Mental Wellbeing Scale (WEMWBS)^59,60^ for measuring mental well-being;
iii. State Anxiety Inventory (STAIX-1)^41,61^ for measuring state anxiety levels;
iv. Mind Wandering Spontaneous (MW-S)^42,62^ for measuring the propensity to spontaneously wander with the mind;
v. Positive and Negative Affect Schedule (PANAS)^43,63^ for measuring positive affect that refers to how energetic, enthusiastic, and alert a person feels and negative affect that is a condition of overall distress;
vi. Difficulties in Emotion Regulation Scale (DERS)^44,64^ for assessing emotional control problems;
vii. Resilience Scale (RS-14)^45,65^ for measuring resilience globally;
viii. and (ix) Attention Control Shifting and Distraction (AC-S and AC-D)^42,62^ for measuring attentional distraction and shifting.

The questionnaires and their psychometric properties are described in detail in the Supplementary Material.

The scores obtained from the questionnaires as measure of the psychological investigated outcomes were used as dependent variables in the statistical models to evaluate the efficacy of our MBI. We also collected at t0 only data from STAI-X2 questionnaires measuring trait anxiety^61^, to be included in the model as covariate. We assumed that the baseline levels of trait anxiety, implicated in the personal tendency to respond with concerns, troubles and worries to various situations^66–68^, could be strongly associated with the outcomes of interest. In addition, empirical studies suggest an inverse relationship between trait mindfulness and trait anxiety, so that people with higher trait anxiety are more likely to have a lower trait mindfulness^69^. Being our intervention built to be easy-to-learn and to produce quick benefits also via an increase of mindfulness, there may be a higher possibility of the observable beneficial effect to be influenced by this trait characteristic. In addition, for each participant, sociodemographic information together with a pool of other variables related to COVID-19 and academic life was collected through a specifically developed background questionnaire administered at baseline only, to describe the sample and to be included as covariates in the statistical model.

For the first cohort, we decided to offer the control group the same intervention straight after the measures were acquired at t1, therefore we were unable to collect the follow-up measures (t2). Regarding the second cohort, the treatment group received the intervention before the summer break, and we offered the control group the same intervention after the summer break. This delay allowed us to collect the follow-up measures on participants from the second cohort, who were asked to fill in the same questionnaires at follow-up, after 3 months from the treatment group receiving the last IM class.

### Statistical analysis

Questionnaires were scored following the provided guidelines for each questionnaire and internal consistency was assessed via Cronbach’s α coefficient^70^.

The two cohorts were analyzed both in their whole (main analysis) and separately (secondary analysis) following an intention-to-treat approach, avoiding bias related to dropouts and participants’ adherence and therefore having a conservative estimation of the treatment effect. Differences between the two groups (treated and control) and between the two cohorts at baseline characteristics, collected via the background questionnaire, were investigated using Wilcoxon test for the non-normally distributed age variable and chi-squared or fisher exact test for the categorical variables. Hedges’s effect size statistic was calculated for each variable by using the cohen.d function in the effsize R package.

Linear Mixed Effect (LME) models^71^ have been applied to evaluate the pre-post treatment changes on each outcome. A random intercept for subjects in the form of 1|subject had been used to adjust the models for intra-subject variability produced by the two repeated measurements at t0 and t1 carried out on the same patients. We estimated the coefficient of the interaction between time and intervention to measure the difference in slopes between the two groups indicating how much more the intervention group is improving over time with respect to the investigated endpoints, compared to the control group over the same period.

In the main analysis, all the models were adjusted for age, sex, previous meditation experience, baseline level of trait anxiety and a variable indicating the cohort to which each subject belongs to. In addition to avoid residual confounding due to covariate misbalances among the two cohorts we create a PS using the background variables resulted to be statistically significant different between the two cohorts (i.e., university, course language, academic course year, housing situation, work, sport activities, feeling stressed due to covid situation, feel competition among students) and we added the PS as covariate in the model. In the secondary analysis all the linear mixed models, as above, were adjusted for age, sex, previous meditation experience, baseline level of trait anxiety and academic course year. For the second cohort only, as follow-up data was available, an analysis fitting the same LME model as above was performed to verify if the effect of our proposed intervention remained the same, improved or worsened during the follow-up period (t2 vs t0).

Normality of residuals for each model was assessed graphically through histograms and following Kim guidelines^72^. Benjamini–Hochberg correction, fixing the False Discovery Rate (FDR) at α 0.05 was used to account for multiple comparisons^73^. A *p*-value ≤ 0.05 on a 2-sided test was considered as statistically significant.

We also performed a multiple mediation post-hoc analysis using lavaan R package^74^ to investigate the direct effect of the treatment on well-being and its indirect effect mediated by the attentional control and emotional regulation.

Descriptive statistics are reported as the means ± standard deviation (SD). All analyses were performed using R 3.5.1 software^75^.

### Ethics statement

The study was approved by the Ethics Committee of the Department of Brain and Behavioral Sciences of the University of Pavia (Prot. n 74/21). The study was performed in accordance with the ethical standards as laid down in the 1964 Declaration of Helsinki and its later amendments. Students were informed that their personal data would be treated in accordance with the privacy policy and all participants signed informed consent.

The study was registered in ClinicalTrials.gov database (Registration Number NCT05567991 on 5/10/2022).

## Supplementary Information

Supplementary Information.

## Data Availability

The dataset for this study can be found in the Supplementary Materials.
