# Supplementary material for: Improving stress management, anxiety, and mental well-being in medical students through an online Mindfulness-Based Intervention: a randomized study
Source: Sci Rep. 2023 May 22;13:8214. doi: 10.1038/s41598-023-35483-z (PMC10201046; doi:10.1038/s41598-023-35483-z)
Supplement: Supplementary file 1 — Supplementary Information. [file 41598_2023_35483_MOESM1_ESM.zip › Supplementary_material_revised/Supplementary Material_revised.pdf]

Improving stress management, anxiety, and mental well-being in medical students through an online Mindfulness-Based Intervention: a randomized study

Supplementary Material

Measures

Each participant in both groups and cohort at the different time points, completed online via Google Forms the Italian validated version of nine questionnaires:

1) The Perceived Stress Scale (PSS)[1] is a 10-items questionnaire that measures the perception of stress and the degree to which situations in one's life are appraised as stressful during the last month. Answers are given on a 5-point Likert scale from "never" to "often" and items were designed to tap how unpredictable, uncontrollable, and overloaded respondents find their lives. Higher scores are associated with a greater stress perception. The Italian version of the PSS proved to be a reliable instrument, as indicated by a Cronbach's alpha of 0.74[2]. The authors of the Italian PSS also reported for the subjects aged ≤ 30 a mean score of 15.9 ± 6.3 (N=80).

2) The Warwick-Edinburgh Mental Wellbeing Scale (WEMWBS)[3] is a 14-items questionnaire of mental well-being including subjective well-being and psychological functioning. All items in a 5-point Likert scale are worded positively and addressed aspects of positive mental health and measure the frequency of the subject's attitudes from "never" to "always". Higher scores indicate mental well-being. The Italian version of the WEMWB proved to be a reliable instrument, as indicated by a Cronbach's alpha that ranged from 0.83 to 0.86[4]. The authors of the Italian WEMWBS reported for the students a mean score of 41.45 ± 6.66 (N=85).

3) State Anxiety Inventory (STAIX-1)[5] questionnaire is a self-report psychological inventory consisting of 20 items rated on a 4-point Likert scale from "not at all" to "very so much". The STAI-X1 assesses a specific form of anxiety which is state anxiety (fear of a specific event). Higher state anxiety levels are positively associated with higher scores. Both the original questionnaire and its Italian version[6] possess good psychometric properties. Julian[7] stated that a cutoff of 39-40 has been indicated to detect clinically significative symptoms.

4) Mind Wandering Spontaneous (MW-S)[8] measures the individual's propensity to spontaneously wander with the mind. For scoring of items 1, 2, and 4, a 7-point Likert scale from 1 which is "rarely" to 7 which is "a lot" and for item 3, from 1 which is "never" to 7 which is "almost always" is used. Higher score indicates a higher tendency of mind wandering in daily life. The psychometric properties of this questionnaire have been tested also in its Italian version, showing a Cronbach's alpha of 0.77[9]**.** The authors of the original version of MW-S reported over a population of Canadian students a mean score of 3.16 ± 0.79 (N=192)[8].

5) Positive and Negative Affect Schedule (PANAS)[10] measures two general dimensions: i) Positive affect (PA) refers to how energetic, enthusiastic, and alert a person feels. The participant has to express how much he/she experience generally each affect in a 5-point-scale from "not at all" to "very much". Low PA is associated with melancholy and lethargy, whereas high PA is associated with great energy, focus, and enjoyment. ii) Negative affect (NA) is a condition of overall distress and unpleasant engagement, with a low NA indicating peace and serenity. The Italian version of the PANAS proved to be a reliable instrument, as indicated by a Cronbach's alpha that ranged from 0.83 to 0.87[11]. The authors of the Italian version of PANAS reported a mean score of 27±7 in the PA subscale while a mean score of 16±6.2 in the NA subscale in a sample of N=600 participants, with a mean age of 27.9±9.78[11].

6) Difficulties in Emotion Regulation Scale (DERS)[12] is one of the most widely used tests for assessing emotional control problems in adults. The 36-item self-report scale is used to generate scores on the following six subscales by asking respondents how they connect to their emotions: *a*) non-acceptance of emotional responses; *b*) difficulty engaging in goal-directed behavior; *c*) impulse control difficulties; *d*) lack of emotional awareness; *e*) Limited access to emotion regulation strategies; and *f*) lack of emotional clarity. A 5-point Likert scale from 1 which is "almost never" to 5 which is "almost always" is used to score each item. Higher score indicates higher difficulties in emotion regulation, in other words, higher emotional dysregulation. The Italian version of DERS used in this study has shown good psychometric properties[13]. The factorial structure of the Italian DERS has been slightly changed from the original one as well as three items were removed. The Cronbach,'s alpha of the Italian DERS has been reported to be 0.90. The authors of the Italian DERS reported a mean total score of 61.38±15.37 among a sample of N=190 participants with a mean age of 30.8±9.7.

7) Resilience Scale (RS-14)[14] is short form represents the short version of the Resilience Scale. It is made up of 14 elements from the Resilience Scale. It has been developed to measure resilience globally. Applicable for different ranges of age groups each item has a 7-point Likert scale ranging from 1 (strongly disagree) to 7 (strongly agree). Higher scores indicate a higher level of resilience. The total of the response values for each item is used to compute the score. The Italian version of the RS-14 proved to be a reliable instrument, as indicated by a Cronbach's alpha of 0.88[15]. The authors of the Italian version of RS-14 also reported a mean score of 76.13±10.48 in the scale among a sample of participants (N=150) composed by 85% of people aged between 18-35 years old[15].

8 and 9) Attentional Control: Shifting and Distraction (AC-S and AC-D)[8] are two questionnaires derived from the Attentional Control Scale developed by Derryberry and Reed[16]. Attentional distraction and Attentional Shifting differences are 2 different factors which Attentional Control Scale have. For the study ACS is separated into 2 different scales (AC-D and AC-S) each having 4 different statements which were rescored to provide consistency. All items on both questionnaires are graded on a 5-point Likert scale, with 1 being *almost never* and 5 being *always*. Higher score for both questionnaires indicates higher attentional distraction and difficulties in attentional shifting. The psychometric properties of this questionnaire have been tested also in its Italian version, indicating a Cronbach’s alfa of 0.83 for AC-S and 0.75 for AC-D[9]. The authors of the original version of AC-S and AC-D reported over a population of Canadian students (N=192) a mean score of 2.76±0.65 for the AC-S scale 3.30±0.79 for the AC-D scale[8].

We also collected at t0 only data from STAI-X2 questionnaires measuring trait anxiety, to be used as covariate in all the fitted models. The STAI-X2[5] is a self-report psychological inventory consisting of 20 items rated on a 4-point Likert scale which assesses trait anxiety (anxiety level as a personal attribute). Trait anxiety levels are positively associated with higher scores. Both the original questionnaire and its Italian version[17] possess good psychometric properties.

References

1. Cohen, S., Kamarck, T. & Mermelstein, R. A global measure of perceived stress. *J. Health Soc. Behav.* **24**, 385–396 (1983).
2. Mondo, M., Sechi, C. & Cabras, C. Psychometric evaluation of three versions of the Italian Perceived Stress Scale. *Curr. Psychol.* 1–9 (2019). doi:10.1007/s12144-019-

0132-8

3. Tennant, R. *et al.* The Warwick-Dinburgh mental well-being scale (WEMWBS): Development and UK validation. *Health Qual. Life Outcomes* **5**, 63 (2007).
4. Gremigni, P. & Stewart-Brown, S. Measuring mental well-being: Italian validation of the Warwick-Edinburgh Mental Well-Being Scale (WEMWBS). *G. Ital. di Psicol.* **38**, 485–505 (2011).
5. Spielberger, C. D. Manual for the State-trait Anxietry, Inventory. *Consult. Psychol.* (1970).
6. Pancheri, P. & Lushene, R. E. STAI : State-trait anxiety inventory : questionario di autovalutazione per l'ansia di stato e di tratto : manuale di istruzioni / di Charles D. Spielberger, Richard L. Gorsuch, Robert E. Lushene ; traduzione ed adattamento a cura di Renato Lazzari, Paolo Pancheri - OPAC - Biblioteca nazionale di Firenze. in *Firenze : OS, c1980 (stampa 1981)*
7. Julian, L. J. Measures of anxiety: State-Trait Anxiety Inventory (STAI), Beck Anxiety Inventory (BAI), and Hospital Anxiety and Depression Scale-Anxiety (HADS-A). *Arthritis Care Res. (Hoboken).* **63 Suppl 1**, (2011).
8. Carriere, J. S. A., Seli, P. & Smilek, D. Wandering in both mind and body: individual differences in mind wandering and inattention predict fidgeting. *Can. J. Exp. Psychol.* **67**, 19–31 (2013).
9. Chiorri, C. & Vannucci, M. Replicability of the psychometric properties of trait-levels measures of spontaneous and deliberate mind wandering. *Eur. J. Psychol. Assess.* **35**, 459–468 (2019).
10. Crawford, J. R. & Henry, J. D. The Positive and Negative Affect Schedule (PANAS): Construct validity, measurement properties and normative data in a large non-clinical sample. *Br. J. Clin. Psychol.* **43**, 245–265 (2004).
11. A, T., RR, M. & Jr, C. P. Factorial and construct validity of the Italian Positive and Negative Affect Schedule (PANAS). *Eur. J. Psychol. Assess.* **19**, 131–141 (2003).
12. Gratz, K. L. & Roemer, L. Multidimensional Assessment of Emotion Regulation and Dysregulation: Development, Factor Structure, and Initial Validation of the Difficulties in Emotion Regulation Scale. *J. Psychopathol. Behav. Assess. 2004 261* **26**, 41–54 (2004).
13. Sighinolfi, C., Pala, A. N., Chiri, L. R., Marchetti, I. & Sica, C. Difficulties in Emotion Regulation Scale (DERS): Traduzione e adattamento Italiano [Difficulties in Emotion Regulation Scale (DERS): The Italian translation and adaptation]. *Psicoter. Cogn. e*

Comport. **16**, 141–170 (2010).

14. Resilience Scale User's Guide | The Resilience Center.
15. Callegari, C. *et al.* Reliability and validity of the Italian version of the 14-item Resilience Scale. *Psychol. Res. Behav. Manag.* **9**, 277 (2016).
16. Derryberry, D. & Reed, M. A. Anxiety-related attentional biases and their regulation by attentional control. *J. Abnorm. Psychol.* **111**, 225–236 (2002).
17. Lazzari, R. & Pancheri, P. Questionario di valutazione dell'ansia di stato e di tratto [State-Trait Anxiety Inventory]. *Organ. Spec. Firenze* (1980).

Supplementary Tables

Supplementary Table 1. Differences at the baseline between the two cohorts

Variable	Mean (SD) 1 cohort (n=239)	Mean (SD) 2 cohort (n=123)	p-value
Age	23.11	22.44	**0.02**
	**N (%)**	**N (%)**	**p-value**
**University**			
Brescia	1 (0.4%)	104 (84%)	**<0.001**
Ferrara	1 (0.4%)	0	
Genova	2 (1%)	0	
Milano	29 (12%)	2 (2%)	
Modena e Reggio Emilia	1 (0.4%)	0	
Parma	15 (6%)	1 (1%)	
Pavia	187 (78%)	15 (12%)	
Roma - Tor Vergata	2 (1%)	1 (1%)	
NA	1 (0.4%)	0	
**Sex**			
Male	57 (24%)	27 (22%)	0.78
Female	182 (76%)	96 (78%)	
Nationality			
Italian	230 (96%)	122 (99%)	0.20
Other	9 (4%)	1 (1%)	
**Course Language**			**<0.001**
Italian	204 (85%)	121 (98%)	
English	35 (15%)	2 (2%)	
**Course Year**			**<0.001**
1	52 (22%)	1 (1%)	
2	10 (4%)	61 (50%)	
3	41 (17%)	42 (34%)	
4	49 (20%)	6 (5%)	
5	30 (13%)	2 (1%)	
6	36 (15%)	7 (6%)	
Supplementary year/s	21 (9%)	4 (3%)	
**In compliance with the exams**			
No, there is problem	54 (23%)	15 (12%)	0.06
No, there is no problem	25 (10%)	15 (12%)	
Yes	159 (66%)	93 (76%)	
NA	1 (0.4%)	0	
**Housing situation (i.e., Where did you live in the last months?)**			
With partner	17 (7%)	1 (1%)	**<0.001**
With family	152 (64%)	107 (87%)	
Alone	15 (6%)	4 (3%)	
Shared apartment	33 (14%)	9 (7%)	
College	17 (7%)	1 (1%)	
Erasmus	3 (1%)	1 (1%)	
NA	2 (1%)	0	
**Work**			
No	213 (89%)	94(76%)	**0.01**
(Part-time)	5 (2%)	4(3%)	
(Full-time)	0	1(1%)	
A couple of days a week	21 (9%)	24(19%)	
**Economic Situation**			
Comfortable	100 (42%)	50 (41%)	0.33
More than Comfortable	56 (23%)	31 (25%)	
Scarce	12 (5%)	12 (10%)	
Sufficient	69 (29%)	30 (24%)	
NA	2 (01%)		

**Sport activities**			
Little or nothing	97 (41%)	43 (34%)	**0.04**
Yes, but irregular	93 (39)	40 (33%)	
Yes, regular	49 (20%)	40 (33%)	
**Number of books read in a year**			
0-1	83 (35%)	43 (35 %)	0.9
2-5	81 (34%)	44 (36 %)	
> 5	75 (31%)	36 (29%)	
**Cultural/sport association membership**			
No	142 (59%)	76 (62%)	0.75
Yes	97 (41%)	47 (38%)	
**Religious**			0.33
No	151 (63%)	70 (57%)	
Yes	88 (37%)	53 (43%)	
**Diet**			0.16
Other	13 (5%)	7 (6%)	
Nutritionist	14 (6%)	10 (8%)	
Allergies / intolerances	4 (2%)	6 (5%)	
None in part.	178 (74%)	92 (75%)	
Vegetarian / vegan	30 (13%)	8 (7%)	
**Sexual satisfaction**			
I do not answer	39 (16%)	31 (25%)	0.14
Unsatisfactory	86 (36%)	42 (34%)	
Satisfying	111 (46%)	50 (40%)	
NA	3 (1%)	0	
**Knowledge about meditation**			
Clear idea	57 (24%)	18 (15%)	0.1
Vague idea	176 (74%)	103 (84%)	
I did not know	6 (2%)	2 (2%)	
**Previous meditation experience**			
Yes	104 (44%)	35 (28%)	**0.007**
No	135 (56%)	88 (72%)	
**Regular sleep**			
Not	76 (32%)	46 (38%)	0.36
Yes	162 (68%)	77 (62%)	
NA	1 (0.4%)	0	
**Disability**			0.28
No	216 (90%)	116 (94%)	
Yes	23 (10%)	7 (6%)	
**Drugs consumption**			0.24
Cigarettes	14 (6%)	12 (10%)	
Alcohol	14 (6%)	5 (4%)	
Psychoactive drugs	11 (5%)	4 (3%)	
Leg Drugs	6 (2%)	0	
Other	2 (1%)	1 (1%)	
A combination	8 (3%)	1 (1%)	
None of the previous	184 (77%)	100 (81%)	
**Substances use**			
Coffee / tea	124 (52%)	87 (70%)	0.13
Supplements	7 (3%)	11 (9%)	
Energy drink	0	2 (2%)	
A combination	36 (15%)	23 (19%)	
NA	72 (30%)	0	
**Do you have support from your inner circle?**			
No	10 (4%)	8 (7%)	0.49
Yes	227 (95%)	115 (93%)	
NA	2 (1%)		
**Feel competition among students**			
No	72 (30%)	52 (42%)	**0.03**
Yes	166 (69%)	71 (58%)	
NA	1 (0.4%)	0	
**Feeling stressed due to academic deadlines**			
Enough	78 (33%)	45 (49%)	0.3
Much	157 (66%)	74 (48%)	
Little or nothing	3 (1%)	4 (3%)	
NA	1 (0.4%)	0	

**Were you negatively affected by COVID-19 pandemic?**			0.21
Enough	149 (62%)	68 (56%)	
Little or nothing	22 (9%)	18 (14%)	
Much	65 (27%)	37 (30%)	
NA	3 (1%)	0	
**Negative effect of COVID-19 pandemic**			
Social relations	68 (28%)	31 (24%)	0.51
Family relationships	1 (0.4%)	0	
Study	4 (2%)	2 (2%)	
A combination	150 (63%)	80 (66%)	
Other	11 (5%)	4 (3%)	
No effect negatively	4 (2%)	6 (5%)	
NA	1 (0.4%)	0	
**Feeling stressed due to COVID-19 situation**			
No	16 (7%)	20 (16%)	**0.008**
Yes	220 (92%)	103 (84%)	
NA	3 (1%)	0	

NA= Missing

Supplementary Table 2. Differences at the baseline characteristics between the two groups in each cohort.

	1 cohort			2 cohort		
**Variable**	**Mean (SD) treatment (n=106)**	**Mean (SD) control (n=133)**	**p-value**	**Mean (SD) treatment (n=68)**	**Mean (SD) control (n=55)**	**p-value**
**Age**	23.31 (4.96)	22.95 (2.78)	0.45	22.03 (1.89)	22.94 (3.97)	0.4
	**N (%)**	**N (%)**		**N (%)**	**N (%)**	
**University**						
Brescia	1 (1%)	0	0.21	59 (87%)	45 (82%)	0.62
Ferrara	0	1 (1%)		0	0	
Genova	2 (2%)	0		0	0	
Milano	16 (15%)	13 (10%)		1 (1%)	1 (2%)	
Modena e Reggio Emilia	1 (1%)	0		0	0	
Parma	6 (6%)	9 (7%)		0	1 (2%)	
Pavia	80 (75%)	107 (80%)		8 (12%)	7 (13%)	
Roma - Tor Vergata	0	2 (1%)		0	1 (2%)	
NA	0	1 (1%)		0	0	
**Sex**						
Male	29 (27%)	28 (21%)	0.26	14 (21%)	13 (24%)	0.68
Female	77 (73%)	105 (79%)		54 (79%)	42 (76%)	
**Nationality**						
Italian	100 (94%)	130 (98%)	0.17	67 (98%)	55 (100%)	1
Other	6 (6%)	3 (2%)		1 (2%)	0	
**Course Language**						
Italian	94 (89%)	110 (83%)	0.27	67 (98%)	54 (98.1%)	1
English	12 (11%)	23 (17%)		1 (2%)	1 (1.9%)	
**Course Year**						
1	22 (21%)	30 (23%)	0.19	0	1 (2%)	0.66
2	5 (5%)	5 (4%)		34 (50%)	27 (49%)	
3	15 (14%)	26 (19%)		26 (38%)	16 (29%)	
4	28 (26%)	21 (16%)		3 (4%)	3 (5%)	
5	13 (12%)	17 (13%)		1 (2%)	1 (2%)	
6	11 (10%)	25 (19%)		2 (3%)	5 (9%)	
Supplementary year/s	12 (11%)	9 (7%)		2 (3%)	2 (4%)	
**In compliance with exams**						
No, there is problem	17 (16%)	37 (28%)	0.10	10 (7%)	5 (7%)	0.54
No, there is no problem	11 (10%)	14 (10%)		7 (5%)	8 (15%)	
Yes	77 (73%)	82 (62%)		51 (38%)	42 (78%)	
NA	1 (1%)	0		0		

**Housing situation**						
With partner	8 (7%)	9 (7%)	0.53	0	1 (2%)	0.46
With family	66 (62%)	86 (65%)		60 (88%)	47 (85%)	
Alone	9 (8%)	6 (4%)		1 (2%)	3 (6%)	
Shared apartment	15 (14%)	18 (14%)		5 (7%)	4 (7%)	
College	7 (7%)	10 (8%)		1 (2%)	0	
Erasmus	0	3 (2%)		1 (2%)	0	
NA	1 (1%)	1 (1%)		0	0	
**Work**						
No	94 (89%)	119 (89%)	0.77	53 (78%)	41 (75%)	0.72
Every day (Part-time)	3 (3%)	2 (1%)		2 (3%)	2 (4%)	
Every day (Full-time)	0 (0%)	0 (0%)		0	1 (2%)	
A couple of days/ week	9 (8%)	12 (9%)		13 (19%)	11 (20%)	
**Economic Situation**						
Comfortable	47 (45%)	53 (40%)	0.79	28 (41%)	22 (40%)	0.74
More than Comfortable	22 (21%)	34 (26%)		17 (25%)	14 (25%)	
Scarce	6 (6%)	6 (4%)		5 (7%)	7 (13%)	
Sufficient	30 (29%)	39 (29%)		18 (26%)	12 (22%)	
NA	1 (1%)	0		0	0	
**Sport activities**						
Little or nothing	39 (37%)	58 (44%)	0.33	24 (35%)	19 (34%)	0.98
Yes, but irregular	41 (39%)	52 (39%)		22 (32%)	18 (33%)	
Yes, regular	26 (24%)	23 (17%)		22 (32%)	18 (33%)	
**N. of books read in a year**						
0-1	37 (35%)	46 (35%)	0.59	27 (40%)	16 (29%)	0.37
2-5	39 (37%)	42 (32%)		21 (30%)	23 (42%)	
> 5	30 (28%)	45 (34%)		20 (29%)	16 (29%)	
**Cultural/sport association membership**						
No	55 (52%)	87 (65%)	0.03	46 (68%)	30 (54%)	0.14
Yes	51 (48%)	46 (35%)		22 (32%)	25 (45%)	
**Religious**						
No	72 (68%)	79 (59%)	0.42	38 (56%)	32 (58%)	0.42
Yes	34 (32%)	54 (41%)		30 (44%)	23 (42%)	
**Diet**						
Other	4 (4%)	9 (7%)	0.58	4 (6%)	3 (5%)	0.84
Nutritionist	5 (5%)	9 (7%)		6 (7%)	4 (7%)	
Allergies /intolerances	1 (1%)	3 (2%)		2 (3%)	4 (7%)	
None in particular	84 (79%)	94 (70%)		52 (76%)	40 (73%)	
Vegetarian / vegan	12 (11%)	18 (13%)		4 (6%)	4 (7%)	
**Sexual satisfaction**						
I prefer to not answer	21 (19%)	21 (16%)	0.54	17 (25%)	14 (25%)	0.77
Unsatisfactory	35 (33%)	51 (39%)		25 (37%)	17 (31%)	
Satisfying	50 (48%)	61 (47%)		26 (38%)	24 (44%)	
**Knowledge about meditation**						
Clear idea	26 (24%)	31 (23%)	0.38	10 (15%)	8 (14%)	0.99
Vague idea	79 (74%)	97 (73%)		57 (84%)	46 (84%)	
I did not know	1 (1%)	5 (4%)		1 (2%)	1 (2%)	
**Previous meditation experience**						
Yes	54 (51%)	81 (61%)	0.12	19 (28%)	39 (71%)	0.89
No	52 (49%)	52 (39%)		49 (72%)	16 (29%)	
**Regular sleep**						
Not	30 (28%)	46 (35%)	0.32	23 (34%)	23 (42%)	0.36
Yes	75 (71%)	87 (65%)		45 (66%)	32 (58%)	
NA	1 (1%)	0		0	0	
**Disability**						
No	99 (94%)	117 (88%)	0.16	63 (93%)	53 (96%)	0.38
Yes	7 (7%)	16 (12%)		5 (7%)	2 (4%)	
**Drugs**						
Cigarettes	7 (7%)	7 (5%)	0.42	7 (10%)	5 (9%)	0.48
Alcohol	9 (8%)	5 (4%)		1 (2%)	4 (7%)	
Psychoactive drugs	4 (4%)	7 (5%)		3 (4%)	1 (2%)	
Smart Drugs	4 (4%)	2 (1%)		0	0	
Other	1 (1%)	1 (1%)		1 (2%)	0	
A combination	5 (5%)	3 (2%)		1 (2%)	0	

None of the previous	76 (72%)	108 (81%)		55 (81%)	45 (82%)	
**Substances**						
Coffee / tea	55 (52%)	69 (92%)	0.67	47 (69%)	40 (73%)	0.31
Supplements	3 (3%)	4 (5%)		8 (12%)	3 (5%)	
Energy drink	0	0 (%)		0	2 (4%)	
A combination	13 (12%)	23 (31%)		13 (19%)	10 (18%)	
NA	35 (33%)	37 (28%)		0	0	
**Do you have support from your inner circle**						
No	7 (7%)	3 (2%)	0.09	4 (6%)	4 (7%)	0.76
Yes	98 (92%)	129 (98%)		64 (94%)	51 (9w%)	
NA	1 (1%)	0		0	0	
**Feel competition among students**						
No	32 (30%)	40 (30%)	0.94	31 (46%)	21 (38%)	0.41
Yes	73 (69%)	93 (70%)		37 (54%)	34 (62%)	
NA	1 (1%)	0		0	0	
**Feeling stressed due to academic deadlines**						
Enough	37 (35%)	41 (31%)	0.73	28 (41%)	17 (31%)	0.5
Much	67 (64%)	90 (68%)		38 (56%)	36 (65%)	
Little or nothing	1 (1%)	2 (1%)		2 (3%)	2 (4%)	
NA	1 (1%)	0		0	0	
**Were you negatively affected by COVID-19 pandemic?**						
Enough	60 (57%)	89 (67%)	0.28	40 (59%)	28 (51%)	0.38
Little or nothing	12 (11%)	10 (7%)		10 (15%)	8 (14%)	
Much	32 (30%)	33 (25%)		18 (26%)	19 (34%)	
NA	2 (2%)	1 (1%)		0		
**Negative effect of COVID-19 pandemic**						
Social relations	35 (33%)	33 (25%)	0.20	18 (26%)	13 (24%)	0.86
Family relationships	1 (1%)	0		0	0	
Study	6 (6%)	5 (4%)		2 (3%)	0	
A combination	61 (57%)	89 (67%)		43 (63%)	37 (67%)	
Other	0	4 (3%)		2 (3%)	2 (4%)	
No effect negatively	2 (2%)	2 (1%)		3 (4%)	3 (5%)	
NA	1 (1%)	0		0	0	
**Feeling stressed due to COVID-19 situation**						
No	6 (6%)	10 (8%)	0.58	12 (18%)	8 (14%)	0.64
Yes	98 (92%)	122 (92%)		56 (82%)	47 (85%)	
NA	2 (2%)	1 (1%)		0	0	

NA= Missing

Supplementary Table 3. Internal consistency for each questionnaire calculated at t0 and t1, separately for each cohort and considering the whole sample.

**Questionnaire**	**Internal Consistency t0**	**Internal Consistency t1**
**STAI-X1**		
All sample	0.95	0.94
1 cohort	0.95	0.94
2 cohort	0.95	0.94
**STAI-X2**		
All sample	0.90	-
1 cohort	0.89	0.91
2 cohort	0.91	0.91
**PSS**		
All sampmple	0.87	0.87
1 cohort	0.86	0.88

2 cohort	0.88	0.88
**WEMWBS**		
All sample	0.88	0.90
1 cohort	0.88	0.91
2 cohort	0.89	0.91
**MW-S**		
All sample	0.84	0.86
1 cohort	0.86	0.88
2 cohort	0.79	0.88
**PANAS *Positive***		
All sample	0.86	0.89
1 cohort	0.85	0.89
2 cohort	0.87	0.89
**PANAS *Negative***		
All sample	0.86	0.88
1 cohort	0.86	0.84
2 cohort	0.86	0.84
**DERS - *All items***		
All sample	0.93	0.94
1 cohort	0.94	0.93
2 cohort	0.93	0.94
**DERS *Lack of acceptance***		
All sample	0.91	0.92
1 cohort	0.91	0.91
2 cohort	0.90	0.91
**DERS *Difficulties in distraction***		
All sample	0.85	0.86
1 cohort	0.85	0.86
2 cohort	0.85	0.86
**DERS *Lack of trust***		
All sample	0.86	0.84
1 cohort	0.86	0.82
2 cohort	0.86	0.82
**DERS *Lack of control***		
All sample	0.88	0.88
1 cohort	0.88	0.90
2 cohort	0.89	0.90
**DERS *Difficulties in recognition***		
All sample	0.84	0.85
1 cohort	0.84	0.83
2 cohort	0.85	0.83
**DERS *Reduced self-awareness***		
All sample	0.86	0.88
1 cohort	0.86	0.91
2 cohort	0.86	0.91
**RS-14**		
All sample	0.86	0.88
1 cohort	0.87	0.88
2 cohort	0.86	0.88
**AC-S**		
All sample	0.91	0.92
1 cohort	0.91	0.92
2 cohort	0.92	0.92
**AC-D**		
All sample	0.76	0.81
1 cohort	0.78	0.75
2 cohort	0.72	0.75
